# Neutralizing Antibody Response following a Third Dose of the mRNA-1273 Vaccine among Cancer Patients

**DOI:** 10.3390/vaccines12010013

**Published:** 2023-12-22

**Authors:** Christopher W. Dukes, Marine Potez, Jeffrey Lancet, Barbara J. Kuter, Junmin Whiting, Qianxing Mo, Brett Leav, Haixing Wang, Julie S. Vanas, Christopher L. Cubitt, Kimberly Isaacs-Soriano, Kayoko Kennedy, Julie Rathwell, Julian Diaz Cobo, Wesley O’Nan, Bradley Sirak, Ning Dong, Elaine Tan, Patrick Hwu, Anna R. Giuliano, Shari Pilon-Thomas

**Affiliations:** 1 Department of Immunology, Moffitt Cancer Center, Tampa, FL 33612, USA; 2 Center for Immunization and Infection Research in Cancer, Moffitt Cancer Center, Tampa, FL 33612, USAanna.giuliano@moffitt.org (A.R.G.); 3 Department of Malignant Hematology, Moffitt Cancer Center, Tampa, FL 33612, USA; 4 Department of Infectious Diseases, Moderna, Inc., Cambridge, MA 02139, USA; 5 Department of Biostatistics and Bioinformatics, Moffitt Cancer Center, Tampa, FL 33612, USA; 6 Immune Monitoring Core Facility, Moffitt Cancer Center, Tampa, FL 33612, USA; 7 Department of Cancer Epidemiology, Moffitt Cancer Center, Tampa, FL 33612, USA; 8 James A. Haley Veterans Hospital, Tampa, FL 33612, USA

**Keywords:** SARS-CoV-2, COVID-19, vaccine, neutralizing antibody, cancer patient, hematologic malignancy, lymphoid cancer

## Abstract

Cancer patients are at an increased risk of morbidity and mortality from SARS-CoV-2 infection and have a decreased immune response to vaccination. We conducted a study measuring both the neutralizing and total antibodies in cancer patients following a third dose of the mRNA-1273 COVID-19 vaccine. Immune responses were measured with an enzyme-linked immunosorbent assay (ELISA) and neutralization assays. Kruskal–Wallis tests were used to evaluate the association between patient characteristics and neutralization geometric mean titers (GMTs), and paired *t*-tests were used to compare the GMTs between different timepoints. Spearman correlation coefficients were calculated to determine the correlation between total antibody and neutralization GMTs. Among 238 adults diagnosed with cancer, a third dose of mRNA-1273 resulted in a 37-fold increase in neutralization GMT 28 days post-vaccination and maintained a 14.6-fold increase at 6 months. Patients with solid tumors or lymphoid cancer had the highest and lowest neutralization GMTs, respectively, at both 28 days and 6 months post-dose 3. While total antibody GMTs in lymphoid patients continued to increase, other cancer types showed decreases in titers between 28 days and 6 months post-dose 3. A strong correlation (*p* < 0.001) was found between total antibody and neutralization GMTs. The third dose of mRNA-1273 was able to elicit a robust neutralizing antibody response in cancer patients, which remained for 6 months after administration. Lymphoid cancer patients can benefit most from this third dose, as it was shown to continue to increase total antibody GMTs 6 months after vaccination.

## 1. Introduction

Cancer patients infected with SARS-CoV-2 are at a higher risk of both morbidity and mortality when compared to the general population [1]. Even after two doses of a COVID-19 vaccine, patients with cancer have lower total antibody geometric mean titers (GMTs) than healthy adults, and the immune response decreases around 6 months following vaccination [2,3,4,5,6,7,8,9,10]. Central to the vaccine-elicited immune response are neutralizing antibodies, which have also been shown to decrease and be insufficient following only two vaccine doses in cancer patients [11,12,13]. The benefit of the third vaccine dose for at-risk populations has been shown via an increase in total antibody GMT [2,14,15,16,17]. These data suggest that a third COVID-19 vaccine dose could increase the levels of neutralizing antibodies and lead to better clinical outcomes for cancer patients.

We previously evaluated the immunogenicity and overall safety of a standard two-dose regimen of the Moderna mRNA-1273 COVID-19 vaccine in cancer patients [2], followed by a third dose of the mRNA-1273 vaccine (100 µg), with immunogenicity results from enzyme-linked immunosorbent assay (ELISA) up to 28 days post-dose 3 (study NCT05054218) [18]. To continue our evaluation, the primary goal of this study was to analyze the neutralizing antibody response pre- and post-dose 3 in the same cohort of patients with solid tumors and hematologic malignancies (HMs). Secondary goals included analyzing the total antibody persistence at 6 months following the third dose, in addition to analyzing a group of patients who participated in both studies to assess the immune response before and after each vaccine dose (Cohort 1). 

## 2. Methods

Details of patient enrollment between 10 September 2021 and 16 December 2021 have been previously published [18]. To assess the duration of antibody response, this cohort of cancer patients was asked to return 6 months following the receipt of their third vaccine dose to have an additional serum sample collected. This study was approved by the Advarra Institutional Review Board (IRB# 00000971) and followed the Declaration of Helsinki. At the time of enrollment, all participants provided written informed consent. Results followed STROBE reporting guidelines (STrengthening the Reporting of OBservational Studies in Epidemiology). 

### 2.1. Study Procedures and Data Collection

To determine the total antibody GMT (IgG) and neutralizing antibody response 6 months post-mRNA-1273 vaccine dose 3, standard phlebotomy practices were followed to collect and process patient blood to cryopreserve serum at −80 °C. Patient information, including sociodemographic characteristics (self-identified by the patients) and medical history, was available from the patient charts [18]. All patient visits took place at Moffitt Cancer Center. 

### 2.2. SARS-CoV-2 Antibody Detection and Quantification Assay

We have previously described the full-length wild-type SPIKE protein ELISA used to assess immunogenicity by measuring seroconversion, the transition from seronegativity (the absence of antibodies against SARS-CoV-2) to seropositivity (the presence of antibodies against SARS-CoV-2) [2,19]. Total antibody GMTs were quantified using the human SARS-CoV-2 serology standard, courtesy of the National Institute of Health. Positive results were sera concentrations above the threshold (three standard deviations plus the average of the negative control sera pool). Sera were tested at Moffitt Cancer Center.

### 2.3. SARS-CoV-2 Neutralizing Antibody Detection and Quantification Assay

Sera to be analyzed for neutralizing antibodies were tested using a previously described pseudovirus neutralization assay (Monogram Biosciences, San Francisco, CA, USA; pseudotyped with SARS-CoV-2 G614 full-length SPIKE protein and packaged with HIV genomic vector, pRTV1.FlucP.CNDO-∆U3, containing a luciferase reporter gene in place of an HIV envelope) to detect and quantify neutralization GMTs [20]. A positive anti-SARS-CoV-2 neutralization was defined as a neutralization titer greater than three times the titer of the same serum tested with the assay specificity control. This specificity control uses a rare avian influenza virus envelope, against which human sera is extremely unlikely to have antibodies. This specificity control is also able to detect non-antibody factors that could inhibit the SARS-CoV-2 pseudovirus and result in false positives. 

### 2.4. Statistical Analysis

Descriptive statistics were used to summarize the patient characteristics. As the primary goal of this study was to evaluate the neutralizing antibody response, antibody-negative patients were given an imputed value halfway between zero and the assay detection limit to enable a quantitative immune response estimation. Total antibody and neutralization GMTs, with 95% confidence intervals, were calculated using log_10_-transformed titers and t-distribution, before transformation back to the original scale. Kruskal–Wallis tests were used to evaluate the association between patient characteristics and neutralization GMTs, while GMTs between different timepoints were compared using paired *t*-tests. Spearman correlation coefficients were calculated to determine the correlation between total antibody and neutralization GMT. Observations with missing data were omitted from analysis. Statistical analyses were completed using SAS, version 9.4 (SAS Institute, Inc., Cary, NC, USA) and R software, version 4.0.2 (R Foundation for Statistical Computing, Vienna, Austria). The statistical significance cutoff was a two-sided *p* < 0.05.

## 3. Results

### 3.1. Patient Characteristics

Patient characteristics for the study population are shown in Table 1. A total of 238 cancer patients who were enrolled and returned for the 6-month timepoint were included, with 104 (43.7%) patients having solid tumor malignancies and 134 (56.3%) patients having HMs (including myeloid cancers, lymphoid cancers, and plasma cell disorders). The study population had a median age of 67 years at the time of third dose administration; there were 107 females (45.0%) and 131 males (55.0%); 17 patients identified as Hispanic (7.1%) and 221 identified as non-Hispanic (92.9%); 9 patients identified as African American (3.8%), 3 identified as Asian (1.3%), 219 identified as White (92.0%), and 7 identified as other (none of the aforementioned races; 2.9%). More than half of the patients were in disease remission (60.1%) and had not received any anticancer therapy within 3 months from dose 3 (54.6%). Only a fraction of patients received small molecule therapy (21.8%) or cellular therapy (16.0%), and even fewer received Bruton’s tyrosine kinase (BTK) inhibitors (2.1%), anti-CD20 antibodies (4.2%), or anti-CD38 antibodies (5.9%).

The characteristics of Cohort 1 (the 111 patients for whom there are sera data at each timepoint) are similar to those of the larger study population (Supplemental Table S1), with 38 (34.2%) having solid tumor malignancies and 73 (65.8%) having HM. Of these patients, 64 (57.7%) were 67 years or younger; 50 were female (45.0%) and 61 (55.0%) were male; 4 identified as Hispanic (3.6%) and 107 identified as non-Hispanic (96.4%); 3 identified as African American (2.7%), 1 identified as Asian (0.9%), 106 identified as White (95.5%), and 1 identified as Other (0.9%). Almost three-quarters of the population were in disease remission (73.9%), and more than half had not received any anticancer therapy within 3 months (55.9%). Only a fraction of Cohort 1 received small molecule therapy (27.0%) or cellular therapy (22.5%), and even fewer received BTK inhibitors (0.9%), anti-CD20 antibodies (4.5%), or anti-CD38 antibodies (9.0%).

### 3.2. Neutralization Response

**Figure 1:**
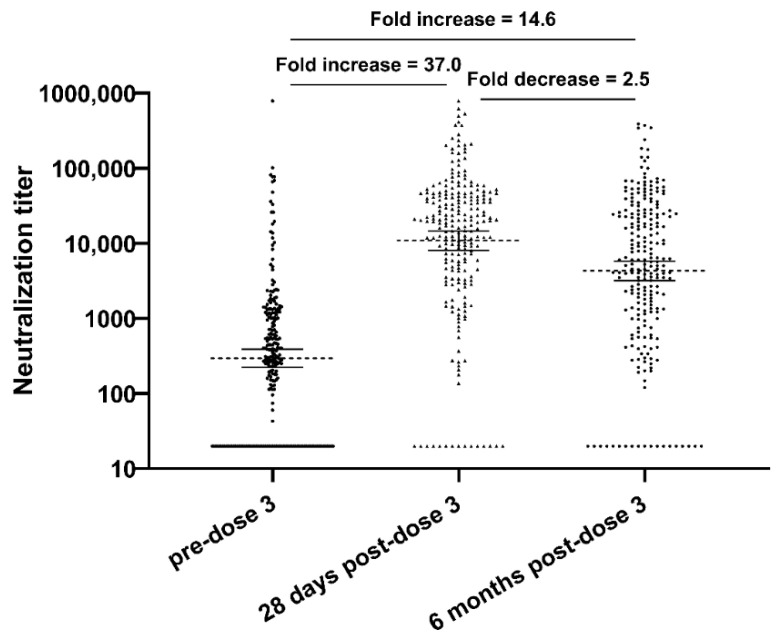
Comparison of neutralization titer at pre-dose 3, 28 days post-dose 3, and 6 months post-dose 3 (*N* = 238; *p* < 0.001).

**Figure 2:**
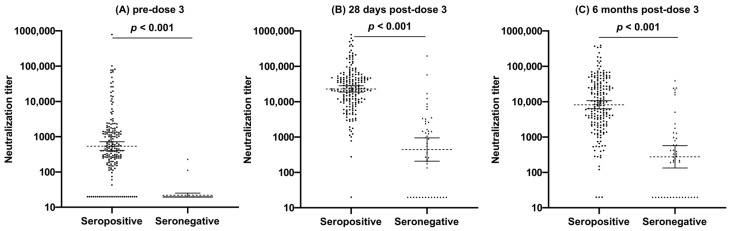
Comparison of neutralization titer at pre-dose 3 (**A**), 28 days post-dose 3 (**B**), and 6 months post-dose 3 (**C**) between pre-dose 3 seropositive and seronegative patients (*N* = 238; 45 seronegative patients and 193 seropositive patients).

Overall, there was a 37-fold increase in neutralization GMT after the administration of the third vaccine dose, followed by a 2.5-fold decrease within 6 months (still a 14.6-fold increase compared to pre-dose 3), with a high variability in neutralization GMT values at each timepoint (Table 2, Figure 1). There were also increases in neutralization GMTs pre-dose 3 for both seropositive (*n* = 193) and seronegative (*n* = 45) patients after the administration of the third vaccine dose, followed by a decrease approximately 6 months after receipt (Figure 2). The neutralization GMTs for pre-dose 3 seropositive patients at pre-dose 3, 28 days post-dose 3, and 6 months post-dose 3 were 539.1 (95% CI 404.1–719.1), 22,949.2 (95% CI 18,725.5–28,125.7), and 8183.6 (95% CI 6304.2–10,623.2), respectively. The neutralization GMTs for pre-dose 3 seronegative patients at pre-dose 3, 28 days post-dose 3, and 6 months post-dose 3 were 22 (95% CI 19.2–25.1), 442.3 (95% CI 207.3–943.8) and 275.9 (95% CI 133.5–570.0) respectively. Of the 45 pre-dose 3 seronegative patients, 33 (73.3%) seroconverted 28 days following the third dose, and all 33 maintained that seropositivity at 6 months. Younger age correlated with a higher vaccine immune response, with a statistically significant increase in neutralization GMT in patients 67 years or younger, compared to patients over 67 years at pre-dose 3 (*p* < 0.001), 28 days post-dose 3 (*p* = 0.049), and 6 months post-dose 3 (*p* = 0.006). Patient cancer type only showed a statistically significant difference in neutralization GMT at 28 days post-dose 3 (*p* = 0.002), with HM patients having a 3.1-fold lower neutralization GMT than solid tumor patients. Patients with lymphoid cancers had the lowest neutralization GMTs at 28 days post-dose 3 among HM patients (*p* = 0.03). Patients with lymphocyte counts above 1 × 10^9^/L had higher neutralization GMTs pre-dose 3, 28 days post-dose 3, and 6 months post-dose 3 (*p* = 0.003, *p* < 0.001, *p* = 0.006, respectively) than patients with lymphocyte counts below 1 × 10^9^/L. 

Patients who received anticancer therapy within 3 months had lower neutralization GMTs pre- and 28 days post-dose 3 (*p* < 0.001) (Table 2). Patients who received small molecules, anti-CD20 antibodies, anti-CD38 antibodies, or BTK inhibitors had lower neutralization GMTs at 28 days post-dose 3 (*p* = 0.001, *p* = 0.002, *p* = 0.03, and *p* = 0.007, respectively); patients who received small molecules and anti-CD38 antibodies started with much lower neutralization GMTs pre-dose 3 (*p* = 0.008 and *p* = 0.02, respectively), and those that received BTK inhibitors maintained a lower neutralization GMT 6 months post-dose 3 (*p* = 0.03). Among the 47 patients with plasma cell disorders, GMTs were positively correlated to total IgG and IgA values (*p* = 0.01).

### 3.3. Correlation between Total Antibody and Neutralization Titers

**Figure 3:**
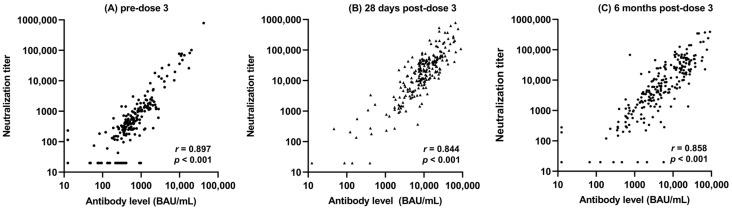
Scatter plot of neutralization titer and total antibody levels at pre-dose 3 (**A**), 28 days post-dose 3 (**B**), and 6 months post-dose 3 (**C**) (*N* = 238).

There was a strong positive correlation between the neutralization GMT and the total antibody GMT at pre-dose 3, 28 days post-dose 3, and 6 months post-dose 3 (Figure 3, Table 3). The largest correlations were observed between neutralization GMT and total antibody GMT at identical timepoints (0.897 pre-dose 3, 0.844 28 days post-dose 3, and 0.858 6 months post-dose 3).

### 3.4. Antibody Response Duration

**Figure 4:**
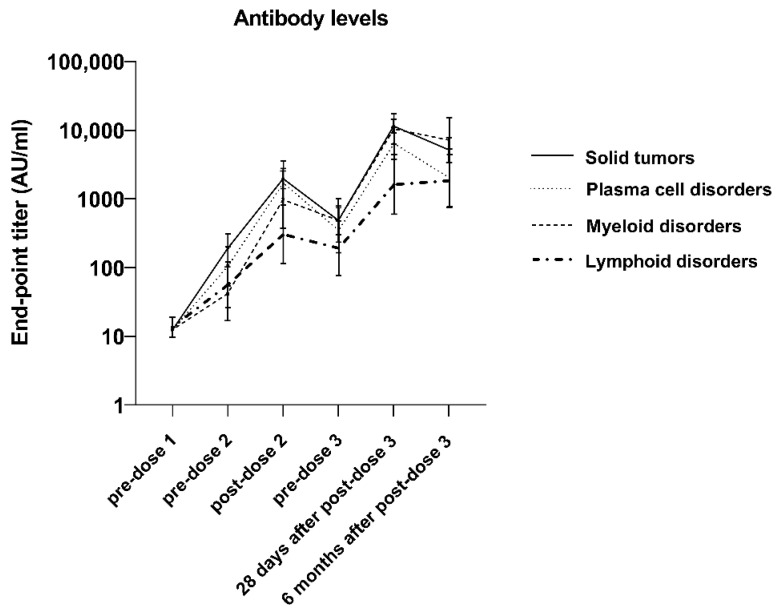
Total antibody titers across timepoints among cancer patients in Cohort 1 (the sub-cohort evaluated after each vaccine dose) (*n* = 111).

Among Cohort 1 (the 111 patients for whom there were sera data at each timepoint), seropositivity increased in all cancer types following the first two vaccine doses, followed by a slight decrease before the third dose. Twenty-eight days after the third dose, all cancer types had >90% seropositivity, except for lymphoid cancer (83.3%). Seropositivity was maintained through 6 months for all except those with plasma cell disorders; plasma cell disorder patients saw a 10.9% decrease in seropositivity, while lymphoid cancer patients saw a 7.1% increase (Supplemental Table S2). There was also an overall 16.7-fold increase in total antibody GMT after the third dose, followed by a 1.8-fold decrease in total antibody GMT between 28 days and 6 months following the third dose. The lymphoid cancer patients, while having the lowest total antibody GMTs among the tumor subtypes, showed a continued 1.1-fold increase between 28 days and 6 months following the third dose (Figure 4, Supplemental Table S3).

## 4. Discussion

This is a continuation of a large study on the mRNA-1273 COVID-19 vaccine third dose administration in cancer patients [18], with data now available from both ELISA and neutralization assays. The data presented herein confirm the earlier results that the administration of a third vaccine dose to cancer patients seems to be very beneficial, in terms of functional immune response and duration, especially for those with lymphoid cancer. Most patients showed a strong neutralizing antibody response to the vaccine 4 weeks following the receipt of dose 3, with titers increasing 14.0- to 57.5-fold, depending on their diagnosed cancer type. While the neutralization GMTs decreased between 28 days and 6 months post-dose 3, they were still 10.2- to 21.9-fold above those recorded pre-dose 3.

We noted several important observations of neutralizing the antibody response after the third vaccine dose. Patients 67 years and younger had a much stronger immune response than those over 67 and maintained that immune response 6 months following the third vaccine dose, likely due to the naturally decreasing humoral immune responses seen in older individuals [21]. Patients with solid tumor malignancies had much stronger immune responses than those with HMs (likely due to B-cell defects [22,23]) 4 weeks after the third dose; however, this statistical difference was lost at 6 months, results that are similar to those published for other mRNA vaccines [24]. Neutralizing antibody responses at 28 days post-dose 3 were reduced in patients who had lower lymphocyte counts (≤1 × 10^9^/L) and those who had received anticancer therapies in the last 3 months, particularly those that received BTK inhibitors, small molecules, anti-CD20 antibodies, and anti-CD38 antibodies (results also noted in other studies) [25,26,27,28]. Most importantly, we observed increased neutralization GMTs, regardless of cancer type or treatment, even in patients that were seronegative prior to dose 3 (similar to the total antibody GMT increase previously described [18]), and at 6 months following the receipt of the third vaccine dose, those titers were still at least 10-fold greater than those recorded prior to dose 3.

We observed a statistically significant correlation between the total antibody GMT and the neutralization GMT at each timepoint, which is in line with the current literature [29]. While we have previously shown that the third vaccine dose elicits a total antibody increase, measured with ELISA, 28 days post-dose 3 [18], we observed a continued increase in total antibody GMT 6 months following dose 3, compared to the pre-dose 3 total antibody GMTs. Finally, in perhaps one of the most important findings of this study, we showed a continued increase in total antibody GMT, as measured with ELISA, for lymphoid patients between 28 days and 6 months post-dose 3, which is extremely important clinically, as these patients consistently have some of the lowest immune responses to the available vaccines [30].

Our study had a few limitations, the first being that we did not determine immunogenicity against different SARS-CoV-2 variants, particularly the Omicron variant, against which existing data show reduced vaccine efficacy [25,31,32]. The number of patients in each subcategory for analysis was lower in some therapies than others, limiting our ability to make conclusions for certain patient subsets. Finally, the administration of the third vaccine dose for our patients was outside of the 28-day window as recommended for the three-dose priming series for immunocompromised individuals (dose 3 was administered between 6.8 and 8.9 months following dose 2; mean 7.4 months), which could result in our observed immune responses differing from those observed in standard clinical practice. This extended window, however, may be an important consideration in evaluating the optimal timing for the third dose.

There were several strengths to our study. Utilizing a neutralization assay allowed us to determine a functional immune response following the third vaccine dose and to show the correlation between results in the ELISA and neutralization assays. Our data showing the increase in neutralizing antibodies following dose 3, particularly in patients who failed to produce neutralizing antibodies after the first two doses, are further supported by other studies [33]. Additionally, our study had a very large sample size and a diverse patient population with different types of cancers, therapies received, and underlying conditions. Finally, we had a large cohort of 111 patients that we were able to follow throughout the entire three-dose vaccine series, out to 6 months following dose 3.

## 5. Conclusions

The results of this study emphasize the importance of a three-dose primary vaccine series for cancer patients, following current recommendations by the Advisory Committee on Immunization Practices (ACIP) [34,35]. This is especially important for HM patients, patients on immunosuppressive therapy, and patients with decreased or absent humoral immunity. With the constantly evolving variants of concern, including the Omicron variant, our data suggest that it could be beneficial for immunocompromised patients to receive an additional fourth or fifth dose of the vaccine, as currently recommended by ACIP. The timing of vaccine administration in relation to cancer therapy is still unknown, as is the interval between additional vaccine doses to optimize the immune response; further research is necessary to determine such and improve the clinical efficacy of additional doses of the mRNA-1273 vaccine in cancer patients.

## Figures and Tables

**Table 1:** Total study population characteristics (*N* = 238).

	All Cancer Patients (*N* = 238)
	*n* (%)
Age group (median age 67 years)	
≤67 years	124 (52.1)
>67 years	114 (47.9)
Gender	
Male	131 (55)
Female	107 (45)
Ethnicity	
Hispanic	17 (7.1)
Non-Hispanic	221 (92.9)
Race	
African American	9 (3.8)
Asian	3 (1.3)
White	219 (92)
Other ^#^	7 (2.9)
Primary patient category	
Hematologic malignancies	134 (56.3)
Myeloid	33 (24.6)
Lymphoid	54 (40.3)
Plasma cell disorders	47 (35.1)
Solid tumors	104 (43.7)
Disease status	
Previously untreated	21 (8.8)
Remission	143 (60.1)
Relapse/refractory/stable disease	74 (31.1)
Lymphocyte count ^a^	
>1 × 10^9^/L	129 (64.2)
≤1 × 10^9^/L	72 (35.8)
Among plasma cell disorders (*n* = 47)	
IgG level ^a^	
<700 mg/dL	26 (56.5)
≥700 mg/dL	20 (43.5)
IgA level ^a^	
<70 mg/dL	24 (52.2)
≥70 mg/dL	22 (47.8)
IgM level ^a^	
<40 mg/dL	36 (78.3)
≥40 mg/dL	10 (21.7)
Received anticancer therapy within 3 months ^b^	
No	130 (54.6)
Yes	108 (45.4)
Small molecules ^c^	
No	186 (78.2)
Yes	52 (21.8)
Anti-CD20 antibodies within 6 months	
No	228 (95.8)
Yes	10 (4.2)
Anti-CD38 antibodies within 6 months	
No	224 (94.1)
Yes	14 (5.9)
Patients treated with cellular therapy	
No	200 (84)
Yes	38 (16)
Patients treated with cellular therapy type	
Allo-HSCT at any time prior to vaccination	19 (50)
Auto-HSCT within the past 24 months	13 (34.2)
CD19 CAR-T at any time prior to vaccination	5 (13.2)
BCMA CAR-T at any time prior to vaccination	1 (2.6)
BTK inhibitors	
No	233 (97.9)
Yes	5 (2.1)
Line of systemic therapy to date	
0	56 (23.5)
1	97 (40.8)
≥2	85 (35.7)

^#^ Other indicates that the patient does not identify as African American, Asian, or White. ^a^ All labs were performed within 3 months prior to the third dose of the vaccine. Of all patients, 15.5% were missing lymphocyte counts. Among those with plasma cell disorders, 2.1% were missing IgG, 2.1% missing IgA, and 2.1% missing IgM. ^b^ For the purpose of this study, anti-androgen and anti-estrogen hormonal therapies were not considered anticancer therapies. ^c^ Small molecules include proteasome inhibitors, pomalidomide, lenalidomide, tyrosine kinase inhibitors, and venetoclax.

**Table 2:** Geometric mean neutralization titers pre-dose 3, 28 days post-dose 3, and 6 months post-dose 3 by cancer patient characteristic and cancer treatment, with 95% confidence intervals (*N* = 238).

	*n*	Pre-Dose 3	*p*-Value *	28 Days Post-Dose 3	*p*-Value **	6 Months Post-Dose 3	*p*-Value ***
Overall	238	294.3 (221.5–391)		10,876.7 (8118.6–14,571.7)		4311 (3187.7–5830.2)	
Age group (median age 67 years)			<0.001		0.049		0.006
≤67	124	445.8 (308.7–643.8)		13,765 (9433.5–20,085.4)		5946.1 (3854–9173.8)	
>67	114	187.3 (122–287.6)		8418.7 (5352.8–13,240.6)		3038.6 (2006.1–4602.6)	
Gender			0.074		0.416		0.196
Male	131	232.9 (160.6–337.7)		10,238.7 (6952.1–15,078.9)		3742.6 (2527.1–5542.8)	
Female	107	392 (252.7–608.1)		11,712.2 (7451.7–18,408.6)		5125.7 (3191.6–8232)	
Ethnicity			0.684		0.582		0.679
Hispanic	17	253.4 (95–675.6)		9121.3 (2731.6–30,458.4)		4904 (1164.2–20,657.7)	
Non-Hispanic	221	297.7 (220.9–401.2)		11,024.9 (8137.3–14,937.3)		4268.5 (3132.3–5816.8)	
Race			0.707		0.319		0.079
African American	9	186.3 (64.8–536.1)		9756.2 (1497.2–63,574.3)		3596.2 (593.4–21,795.3)	
Asian	3	315.8 (0.8–119,688.4)		37,742.6 (4145.2–343,651.3)		1652.5 (0–353,348,110.9)	
White	219	291.1 (215.2–393.7)		10,275.8 (7561.4–13,964.5)		4106.5 (3011.5–5599.6)	
Other ^#^	7	726.7 (143.6–3677)		43,432.2 (8454.6–223,116.2)		37,547.5 (9493.9–148,496.6)	
Primary patient category ^a^			0.186		0.002		0.184
Hematologic malignancies ^b^	134	251.4 (168.3–375.6)	0.608	6593.8 (4176.4–10,410.6)	0.029	3330.9 (2130.9–5206.6)	0.165
Myeloid	33	319.5 (146–699.1)		13,485.4 (5804.7–31,328.9)		7010.9 (3089–15,912.4)	
Lymphoid	54	212.2 (105.5–426.8)		2966.6 (1263.0–6967.8)		2174.4 (1023.5–4619.5)	
Plasma cell disorders	47	258.2 (134.5–495.7)		9988.9 (5490.6–18,172.6)		3224.3 (1512.2–6874.6)	
Solid tumors	104	360.5 (242.1–537)		20,727.9 (15,638–27,474.4)		6010.5 (4106.6–8797)	
Disease status			0.160		0.913		0.753
Previously untreated	21	361.6 (116.1–1126.2)		15,220.6 (6170.9–37,542.0)		3736.5 (1326.6–10,524)	
Remission	143	341.4 (238.3–489.1)		11,226.4 (7805.7–16,146.1)		4214.7 (2848.6–6235.9)	
Relapse/refractory/stable disease	74	208.4 (124.2–349.6)		9300.8 (5154.2–16,783.4)		4690 (2686–8188.9)	
Lymphocyte count ^c^			0.003		<0.001		0.006
>1 × 10^9^/L	129	398.9 (269.5–590.5)		18,435.1 (13,000.9–26,140.9)		6158.8 (4320.7–8778.8)	
≤1 × 10^9^/L	72	158 (93.2–267.8)		3299.5 (1745.7–6236.2)		1806.9 (930.9–3507.2)	
Among plasma cell disorders (*n* = 47)							
IgG level ^c^			0.289		0.012		0.506
<700 mg/dL	26	181.3 (77.8–422.5)		5359.3 (2377.0–12,083.7)		2683.3 (946.0–7611.3)	
≥700 mg/dL	20	374.4 (122.4–1145)		21,475.2 (8904.1–51,795.1)		3915.7 (1114.9–13,752.7)	
IgA level ^c^			0.165		0.014		0.230
<70 mg/dL	24	171.8 (58.6–503.9)		4491.7 (1731.0–11,655.3)		1953.3 (627.8–6077.9)	
≥70 mg/dL	22	371.7 (167.6–824.7)		22,951.3 (12,215.8–43,121.3)		5349.7 (1796.4–15,931.3)	
IgM level ^c^			0.290		0.107		0.957
<40 mg/dL	36	216.3 (103.7–451.4)		8591.7 (4635.3–15,924.9)		3258.7 (1436.8–7391)	
≥40 mg/dL	10	409.6 (69.6–2412.6)		15,735.4 (2109.4–11,7382.5)		2839.2 (261.9–30,777.6)	
Received anticancer therapy within 3 months ^d^			<0.001		<0.001		0.051
No	130	480.1 (328.2–702.2)		17,528.3 (12,547.1–24,487.1)		6116.3 (4224.8–8854.7)	
Yes	108	163.3 (108.8–245.2)		6124.0 (3760.9–9972.1)		2829.6 (1735.3–4613.9)	
Small molecules ^e^			0.008		0.001		0.140
No	186	366.6 (265.6–505.8)		13,381.5 (9655.7–18,545)		4946.4 (3549.5–6893.0)	
Yes	52	134.2 (75.7–237.9)		5182.5 (2747.0–9777.5)		2636.3 (1291.8–5380.1)	
Anti-CD20 antibodies			0.081		0.002		0.862
No	228	309.3 (232.2–411.9)		12,511.5 (9458.2–16,550.5)		4380.0 (3224.2–5950.1)	
Yes	10	94.9 (13.6–660.8)		446.7 (44.6–4476.8)		3001.1 (396.3–22,723.4)	
Anti-CD38 antibodies			0.023		0.030		0.275
No	224	315.3 (236.6–420.2)		11,356.0 (8359.2–15,427.3)		4527.3 (3325.1–6164.1)	
Yes	14	97.7 (20.7–460.3)		5455.1 (2316.3–12,847.4)		1969.7 (429.9–9024.1)	
Patients treated with cellular therapy			0.379		0.554		0.147
No	200	282.7 (208.9–382.5)		11,397.5 (8452.1–15,369.3)		4024.2 (2918.6–5548.6)	
Yes	38	363.9 (158.7–834.5)		8503.1 (3207.5–22,542.2)		6193.6 (2580.5–14,865.6)	
Patients treated with cellular therapy type							
Allo-HSCT at any time prior to vaccination	19	479.1 (153.4–1496.1)		13,973 (3385.5–57,669.7)		17,795.9 (6538.5–48,435.1)	
Auto-HSCT within the past 24 months	13	402.4 (83.5–1939.9)		19,059.3 (7203.1–50,430.6)		6203.8 (1915.5–20,093)	
CD19 CAR-T at any time prior to vaccination	5	83.9 (1.6–4491.1)		128.8 (3–5542.8)		245.9 (3–20,446.3)	
BCMA CAR-T at any time prior to vaccination	1	816 (.)		23,603 (.)		120 (.)	
BTK inhibitors			0.145		0.007		0.032
No	233	303.7 (227.8–404.9)		11,467.8 (8548.4–15,384.2)		4514.3 (3332.5–6115.4)	
Yes	5	68.0 (8.5–544.1)		923.6 (128.3–6649.2)		503.3 (36.5–6941.0)	
Line of systemic therapy to date			0.205		0.907		0.232
0	56	428.5 (230.8–795.6)		14,685.2 (8390.2–25,703.1)		4029.5 (2198.9–7384.2)	
1	97	310.4 (204.3–471.7)		10,684.9 (6897.9–16,551.2)		6056.9 (3974.6–9230.1)	
≥2	85	216.2 (131.2–356.2)		9107.8 (5264.5–15,756.7)		3057.6 (1716.1–5447.6)	

* Calculated *p* values are comparing patients on a specific therapy with those not on that therapy at pre-dose 3 using Kruskal–Wallis test. ** Calculated *p* values are comparing patients on a specific therapy with those not on that therapy at 28 days post-dose 3 using Kruskal–Wallis test. *** Calculated *p* values are comparing patients on a specific therapy with those not on that therapy at 6 months post-dose 3 using Kruskal–Wallis test. ^#^ Other indicates that the patient does not identify as African American, Asian, or White. ^a^ Calculated *p* values compare hematologic malignancies with solid tumors. ^b^ Calculated *p* values compare myeloid, lymphoid, and plasma cell disorders. ^c^ All labs were performed within 3 months prior to the third vaccine dose. Of all patients, 15.5% were missing lymphocyte counts. Among patients that had plasma cell disorder, 2.1% were missing IgG, 2.1% missing IgA, and 2.1% missing IgM. ^d^ For the purpose of this study, anti-androgen and anti-estrogen hormonal therapies were not considered anticancer therapies. ^e^ Small molecules include proteasome inhibitors, pomalidomide, lenalidomide, tyrosine kinase inhibitors, and venetoclax. Abbreviations: auto-HSCT, autologous hematopoietic stem cell transplantation; allo-HSCT, allogeneic hematopoietic stem cell transplantation; BTK, Bruton’s tyrosine kinase; CAR-T, chimeric antigen receptor T-cell therapy.

**Table 3:** Spearman correlation coefficients of neutralization and total antibody titers pre-, 28 days post-, and 6 months post-dose 3 (*N* = 238).

	Neutralization Titer at Different Timepoints					
	Pre-Dose 3		28 Days Post-Dose 3		6 Months Post-Dose 3	
Antibody Level	Spearman Correlation Coefficients (95% CI)	*p* Value	Spearman Correlation Coefficients (95% CI)	*p* Value	Spearman Correlation Coefficients (95% CI)	*p* Value
Pre-dose 3 (*n* = 238)	0.897 (0.868–0.919)	<0.001	0.662 (0.583–0.727)	<0.001	0.494 (0.390–0.583)	<0.001
28 days post-dose 3 (*n* = 238)	0.515 (0.414–0.602)	<0.001	0.844 (0.802–0.876)	<0.001	0.555 (0.459–0.636)	<0.001
6 months post-dose 3 (*n* = 234)	0.424 (0.312–0.523)	<0.001	0.613 (0.525–0.687)	<0.001	0.858 (0.819–0.888)	<0.001

## Data Availability

The data underlying this article are property of the Moffitt Cancer Center. Data will be shared on request to the corresponding author with permission from the Moffitt Cancer Center.

## Supplementary Materials

The following supporting information can be downloaded at: https://www.mdpi.com/article/10.3390/vaccines12010013/s1, Table S1: Patient characteristics of Cohort 1 (*n* = 111); Table S2: Percent of Cohort 1 patients seropositive for SARS-CoV-2 antibody (95% CI) at each timepoint, as measured with ELISA by tumor type (*n* = 111); Table S3: Total antibody geometric mean titer (AU/mL; 95% CI) of Cohort 1 patients at each timepoint as measured with ELISA by tumor type (*n* = 111).
