# Supplementary material for: Neutralizing Antibody Response following a Third Dose of the mRNA-1273 Vaccine among Cancer Patients
Source: Vaccines (Basel). 2023 Dec 22;12(1):13. doi: 10.3390/vaccines12010013 (PMC10818923; doi:10.3390/vaccines12010013)
Supplement: Supplementary file 1 [file vaccines-12-00013-s001.zip › vaccines-2711799-supplementary/Supplemental Table S3_Neutralizing antibody response.docx]

## Supplemental Table S3. Total antibody geometric mean titer (AU/mL; 95% CI^*^) of Cohort 1 patients at each timepoint as measured by ELISA by tumor type (*n*=111)

|  | Pre-dose 1 | Post-dose 1 | Post-dose 2 | Pre-dose 3 | 28 days post-dose 3 | 6 months post-dose 3 |
| --- | --- | --- | --- | --- | --- | --- |
| Overall | 13.0 (12-14.2) | 96.3 (69.0-134.5) | 1060.5 (730.6-1539.5) | 358.4 (252.6-508.3) | 5988.8 (4305.1-8331.0) | 3401.5 (2353.0-4917.2) |
| Tumor type |  |  |  |  |  |  |
| Hematologic malignancies (*n*=73) | 13.3 (11.7-15.2) | 67.0 (43.6-103.0) | 768.3 (452.6-1304.3) | 305.7 (190.7-489.9) | 4267.0 (2659.0-6847.3) | 2727.5 (1625.8-4575.6) |
| Myeloid (*n*=18) | 12.5 (.) | 41.7 (17-102.1) | 971.0 (371.1-2540.2) | 484.6 (234.0-1003.7) | 10297.7 (6184.9-17145.5) | 7103.0 (3352.6-15048.8) |
| Lymphoid (*n*=29) | 14.7 (10.5-20.5) | 60.5 (28.0-130.6) | 327.9 (122.8-875.8) | 204.9 (81.8-512.9) | 1733.1 (639.5-4696.8) | 1972.6 (822.5-4730.8) |
| Plasma cell disorders (*n*=26) | 12.5 (.) | 104.4 (54.3-200.6) | 1689 (813.1-3508.5) | 347.2 (164.0-734.7) | 6334.0 (3705.2-10827.9) | 1993.1 (751.0-5289.9) |
| Solid tumor (*n*=38) | 12.5 (.) | 193.1 (121.2-307.6) | 1970 (1404.6-2763) | 486.4 (300.0-788.6) | 11485.6 (9108.8-14482.6) | 5168.8 (3398.4-7861.5) |

^*^ Antibody levels below the level of detection were assigned a value of 12.5. The 95% CI cannot be calculated if they are the same values.
