# Supplementary material for: Neutralizing Antibody Response following a Third Dose of the mRNA-1273 Vaccine among Cancer Patients
Source: Vaccines (Basel). 2023 Dec 22;12(1):13. doi: 10.3390/vaccines12010013 (PMC10818923; doi:10.3390/vaccines12010013)
Supplement: Supplementary file 1 [file vaccines-12-00013-s001.zip › vaccines-2711799-supplementary/Supplemental Table S2_Neutralizing antibody response.docx]

## Supplemental Table S2. Percent of Cohort 1 patients seropositive for SARS-CoV-2 antibody (95% CI) at each timepoint as measured by ELISA by tumor type among patients (*n*=111)

|  | Pre-dose 1 | Post-dose 1 | Post-dose 2 | Pre-dose 3 | 28 days post-dose 3 | 6 months post-dose 3 |
| --- | --- | --- | --- | --- | --- | --- |
| Overall | 0.4 (0 - 2.3) | 70.3 (60.9 - 78.6) | 91 (84.1 - 95.6) | 81.1 (75.5 - 85.9) | 95.0 (91.4 - 97.4) | 94.4 (90.7 - 97) |
| Tumor type |  |  |  |  |  |  |
| Hematologic malignancies (*n*=73) | 0.7 (0 - 4.1) | 57.5 (45.4 – 69.0) | 86.3 (76.2 - 93.2) | 74.6 (66.4 - 81.7) | 91.8 (85.8 - 95.8) | 90.8 (84.5 - 95.2) |
| Myeloid (*n*=18) | 0 | 38.9 (17.3 - 64.3) | 100 (81.5 - 100) | 84.8 (68.1 - 94.9) | 97.0 (84.2 - 99.9) | 97.0 (84.2 - 99.9) |
| Lymphoid (*n*=29) | 1.9 (0 - 9.9) | 55.2 (35.7 - 73.6) | 69.0 (49.2 - 84.7) | 61.1 (46.9 - 74.1) | 83.3 (70.7 - 92.1) | 90.4 (79.0 - 96.8) |
| Plasma cell disorders (*n*=26) | 0 | 73.1 (52.2 - 88.4) | 96.2 (80.4 - 99.9) | 83.0 (69.2 - 92.4) | 97.9 (88.7 - 99.9) | 87.0 (73.7 - 95.1) |
| Solid tumor (*n*=38) | 0 | 94.7 (82.3 - 99.4) | 100 (90.7 - 100) | 89.4 (81.9 - 94.6) | 99.0 (94.8 - 100) | 99.0 (94.7 - 100) |
