# Supplementary material for: Neutralizing Antibody Response following a Third Dose of the mRNA-1273 Vaccine among Cancer Patients
Source: Vaccines (Basel). 2023 Dec 22;12(1):13. doi: 10.3390/vaccines12010013 (PMC10818923; doi:10.3390/vaccines12010013)
Supplement: Supplementary file 1 [file vaccines-12-00013-s001.zip › vaccines-2711799-supplementary/Supplemental Table S1_Neutralizing antibody response.docx]

## Supplemental Table S1. Patient characteristics of Cohort 1 (*n*=111)

|  | Cohort 1 sub-population ^a^(*n*=111) |
| --- | --- |
|  | *n* (%) |
| Age group (median age 67 years) | |
| ≤67 years | 64 (57.7) |
| >67 years | 47 (42.3) |
| Gender | |
| Male | 61 (55) |
| Female | 50 (45) |
| Ethnicity | |
| Hispanic | 4 (3.6) |
| Non-Hispanic | 107 (96.4) |
| Race | |
| African American | 3 (2.7) |
| Asian | 1 (0.9) |
| White | 106 (95.5) |
| Other ^#^ | 1 (0.9) |
| Primary patient category | |
| Hematologic malignancies | 73 (65.8) |
| Myeloid | 18 (24.7) |
| Lymphoid | 29 (39.7) |
| Plasma cell disorders | 26 (35.6) |
| Solid tumors | 38 (34.2) |
| Disease status | |
| Previously untreated | 6 (5.4) |
| Remission | 82 (73.9) |
| Relapse/refractory/stable disease | 23 (20.7) |
| Lymphocyte count ^b^ | |
| >1 x 10^9^/L | 55 (63.2) |
| ≤1 x 10^9^/L | 32 (36.8) |
| Among plasma cell disorders (n=47) | |
| IgG level ^b^ | |
| < 700 mg/dL | 15 (60) |
| ≥700 mg/dL | 10 (40) |
| IgA level ^b^ | |
| <70 mg/dL | 13 (52) |
| ≥70 mg/dL | 12 (48) |
| IgM level ^b^ | |
| < 40 mg/dL | 18 (72) |
| ≥40 mg/dL | 7 (28) |
| Received anticancer therapy within 3 months ^c^ | |
| No | 62 (55.9) |
| Yes | 49 (44.1) |
| Small molecules ^d^ | |
| No | 81 (73) |
| Yes | 30 (27) |
| Anti-CD20 antibodies within 6 months | |
| No | 106 (95.5) |
| Yes | 5 (4.5) |
| Anti-CD38 antibodies within 6 months | |
| No | 101 (91) |
| Yes | 10 (9) |
| Patients treated with cellular therapy | |
| No | 86 (77.5) |
| Yes | 25 (22.5) |
| Patients treated with cellular therapy type | |
| Allo-HSCT any time prior to vaccination | 13 (52) |
| Auto-HSCT within the past 24 months | 8 (32) |
| CD19 CAR-T any time prior to vaccination | 3 (12) |
| BCMA CAR-T any time prior to vaccination | 1 (4) |
| BTK inhibitors | |
| No | 110 (99.1) |
| Yes | 1 (0.9) |
| Line of systemic therapy to date | |
| 0 | 20 (18) |
| 1 | 46 (41.4) |
| ≥2 | 45 (40.5) |

^#^ Other indicates the patient does not identify as African American, Asian, or White.

^a^ Three dose cohort includes the subset of patients who had safety and immunogenicity data after doses 1, 2, and 3.

^b^ All labs were within 3 months prior to the third dose of vaccine. 15.5% of all patients were missing lymphocyte count. Among those with plasma cell disorders, 2.1% were missing IgG, 2.1% missing IgA, and 2.1% missing IgM.

^c^ For the purposes of this study, anti-androgen and anti-oestrogen hormonal therapies were not considered anticancer therapy.

^d^ Small molecules include proteasome inhibitors, pomalidomide, lenalidomide, tyrosine kinase inhibitors, and venetoclax.
